# Assessment of the Fertilization Capacity of the Aquaculture Sediment for Wheat Grass as Sustainable Alternative Use

**DOI:** 10.3390/plants11050634

**Published:** 2022-02-25

**Authors:** Marian Burducea, Andrei Lobiuc, Lenuta Dirvariu, Eugen Oprea, Stefan Mihaita Olaru, Gabriel-Ciprian Teliban, Vasile Stoleru, Vlad Andrei Poghirc, Irina Gabriela Cara, Manuela Filip, Mariana Rusu, Valtcho D. Zheljazkov, Cristian-Alin Barbacariu

**Affiliations:** 1 Research and Development Station for Aquaculture and Aquatic Ecology, “Alexandru Ioan Cuza” University, Carol I, 20A, 700505 Iasi, Romania; dirvariu.lenuta@gmail.com (L.D.); eugen.oprea@uaic.ro (E.O.); alin.barbacariu@uaic.ro (C.-A.B.); 2 Human Health and Development Department, “Stefan Cel Mare” University, Strada Universitatii, 720229 Suceava, Romania; andrei.lobiuc@usm.ro; 3 Faculty of Biology, “Alexandru Ioan Cuza” University, Carol I, 20A, 700505 Iasi, Romania; stefan.olaru93@gmail.com; 4 Faculty of Horticulture, “Ion Ionescu de la Brad” Iasi University of Life Sciences, Aleea Mihail Sadoveanu 3, 700490 Iasi, Romania; gabrielteliban@uaiasi.ro (G.-C.T.); vstoleru@uaiasi.ro (V.S.); 5 Research Institute for Agriculture and Environment, “Ion Ionescu de la Brad” Iasi University of Life Sciences, 9 Mihail Sadoveanu Alley, 700789 Iasi, Romania; vlad.poghirc@uaiasi.ro (V.A.P.); carairina@uaiasi.ro (I.G.C.); filipmanuela@yahoo.com (M.F.); rusumariana910@yahoo.com (M.R.); 6 Crop and Soil Science Department, Oregon State University, 109 Crop Science Building, 3050 SW Campus Way, Corvallis, OR 97331, USA; valtcho.jeliazkov@oregonstate.edu

**Keywords:** aquaculture pond sediment, recovery, *Triticum aestivum*, photosynthesis, heavy metals, chlorophyll fluorescence, wheat grass juice quality

## Abstract

Periodic removal of sediment from aquaculture ponds is practiced to maintain their productivity and animal welfare. The recovery of sediment as a plant fertilizer could alleviate the costs of sediment removal. The objective of this study was to test the effects of a dried sediment, extracted from an aquaculture pond used for common carp cultivation, on the growth and physiology of potted wheat grass and the quality of the juice obtained from wheat grass. The results showed that sediment application did not produce significant morphological changes, although the values for plant height (16.94–19.22 cm), leaf area (19.67–139.21 mm^2^), and biomass (3.39–4.26 g/plant) were higher in sediment-grown plants. However, at a physiological level, the effect was negative, decreasing photosynthesis (0.82–1.66 μmol CO_2_ m^2^s^−1^), fluorescence ΦPSII (0.737–0.782), and chlorophyll content (1.40–1.83 CCI). The juice yield was reduced in the sediment treatments (46–58 g/100 g), while the quality was improved by increasing the content of phenols (2.55–3.39 µg/mL gallic acid equivalent), flavonoids (1.41–1.85 µg/mL quercetin equivalent), and antioxidant activity (47.99–62.7% inhibition of; 2,2-diphenyl-1-picrylhydrazyl). The positive results obtained in this study can be attributed to the moderate nutrient content of the sediment and a negligible concentration of heavy metals.

## 1. Introduction

Aquaculture pond sediment is a mixture of uneaten feed and fish feces, with decaying plant, animal, and microorganisms remains. The accumulation of large amounts of sediment has negative effects on aquaculture economic efficiency, as well as on fish health, mainly because of the reduction of pond depth and living space for fish. Moreover, the accumulation of sediment causes a decrease in the concentration of dissolved oxygen due to its consumption by nitrifying bacteria, as well as the production of toxic compounds, such as H_2_S and NO_2_ [1]. Therefore, periodic clearing of the aquaculture ponds is needed in order to avoid these issues. Depending on the type of aquaculture, cleaning of pond sediments can take place several times a year as is the case in organic aquaculture or once every several years, as is the case of aquaculture practiced in natural water bodies [2].

Estimates show that the world aquaculture production will increase by 32% by 2030 [3], thus the continuous development of the aquaculture sector determines a high sediment production. For this reason, there is a need for efficient fish pond sediment management strategies that include both their removal and recovery [4]. This is especially important as uncontrolled spreading of sediment on the field, a widespread practice, can lead to soil pollution with nitrates and water eutrophication [5]. Moreover, leachate can reach groundwater and drinking water sources, threatening human health [6,7].

The aquaculture sediment reuse in agriculture has multiple benefits. The use of sediment in agriculture to fertilize crops and improve soil quality is possible due to the high content of nutrients and organic matter [8]. Previous studies demonstrated the usefulness of sediment application in agriculture [9,10,11]. For example, sediment from pangasius (*Pangasianodon hypophthalmus*) mixed with organic amendments from rice straw could be a good fertilizer for cucumbers (*Cucumis sativus* L.) [11], while fish sediments from organic aquaculture have been shown to be beneficial for white beans [2]. However, when considering the use of sediment in agriculture, physico-chemical analyses must be performed due to the fact that their composition varies depending on the type of aquaculture, the cultivated fish species and its age, the feed used, etc. [12]. According to Dróżdż et al. 2020 [1], the pH of aquaculture sediments can vary between 4 and 7, the humus content between 0.76 and 3.2 T/ha, N between 1.08 and 7.03 g/kg, *p* between 0.22–2.07 g/kg, and K between 0.62–2.25 g/kg.

Commune carp (*Cyprinus carpio*) is a very popular species in Eastern Europe and Asia, being cultivated mainly in natural fresh water bodies or in constructed ponds. According to the Romanian National Strategy for the Fishing Sector 2014–2020, aquaculture is practiced on a total area of 89,615.23 ha of production farms and 8617.55 ha of nurseries. The structure by species is dominated by Asian cyprinids, 32%, and Romanian carp, 31.8%, followed by crucian at 12.56%, trout at 20.45%, and pike, perch, catfish, and sturgeon at 3.19% [13,14,15]. Pond aquaculture generates large amounts of sediments, and their recovery as a plant fertilizer source can be a sustainable solution, in accordance with the new environmental policies, such as the Green Deal [16].

Wheat plants *Triticum aestivum* Linn. are generally used as a model plant to test the effect of different substrates or pollutants [17]. Moreover, wheat grass is used to produce wheat grass juice (WGJ), which is an extract made from young (10–15 cm) wheat plants [18]. The WGJ is a solution with a complex chemical composition, rich in minerals, enzymes, vitamins, phenolics, and chlorophylls, with considerable bioactivities [19]. In general, the use of sediments for plant fertilization has positive effects due to the rich content of nutrients and organic matter. However, depending on their origin, they can cause physiological disturbances, such as decreased photosynthesis, which can reduce plant growth rate and can even cause plant death if the content of heavy metals or pathogens are too high [20]. Under these conditions, when sediment is used in agriculture, its quality must meet certain requirements. The objective of this study was to evaluate the potential recovery of sediment from a commune carp pond aquaculture as a growing substrate for wheat, by assessing the effects on wheat grass production and physiology and the quality of wheat grass juice. Furthermore, the quality of the leachate in terms of nitrogen compounds was evaluated to determine the impact of the use of sediment on the environment.

## 2. Results

### 2.1. Sediment Composition

This study assessed the potential use of aquaculture sediment as a nutrient substrate for wheat grass. The chemical characterization of the sediment is presented in Table 1. Sun-dried sediment had a slightly alkaline pH (7.67–8.06). In terms of nutrients, humus and total N were significantly higher in CS3, while the content of organic matter, *p* and K are significantly higher in CS1 (*p* ˂ 0.05). The concentrations of heavy metals were below the maximum allowable limit for use in agriculture according to the Romanian legislation OM 344/2004 (Cu 500 mg/kg, Zn 2000 mg/kg, Pb 300 mg/kg, and Cd 10 mg/kg). The total coliforms ranged from 760 cfu/g to 1536 cfu/g while *E. coli* was not detected.

### 2.2. Morphology of Wheat Grass and Yield of Wheat Grass Juice

In this study, mixtures (*v*/*v*) of sediment from the three collection stations (CS1, CS2, and CS3) with commercial peat (CP) were performed, resulting in 10 treatments: V1 (CP 100%, control); V2 (CS1 50% + CP 50%); V3 (CS1 75% + CP 25%); V4 (CS1 100%); V5 (CS2 50% + CP 50%); V6 (CS2 75% + CP 25%); V7 (CS2 100%); V8 (CS3 50% + CP 50%); V9 (CS3 75% + CP 25%); and V10 (CS3 100%).

The morphological parameters of wheat grass and the yield of wheat grass juice grown on aquaculture sediment are shown in Table 2. In general, wheat plants have grown uniformly in all treatments, with values of plant height between 16.9 cm and 19.2 cm, and the highest increase compared with the control being in the V4 variant. Leaf area and plant biomass increased the most in the V6 variant compared to V1 (control) (2.6% and 8%, respectively), however without statistical significance. In contrast, in all variants wheat grass juice yield decreased significantly (*p* < 0.05) up to 20% at V10 compared with V1.

### 2.3. Color of Wheat Grass

The color of wheat grass is presented in Table 3. The parameters L (lightness) and b (yellowness) did not vary significantly. The a (greenness) parameter was significantly higher in V7 and V10 than in V4, which means that V4 had a more pronounced green color. There were no significant differences between the V1 (control) variant and the rest of the variants.

### 2.4. Physiology of Wheat Grass

**Figure 1:**
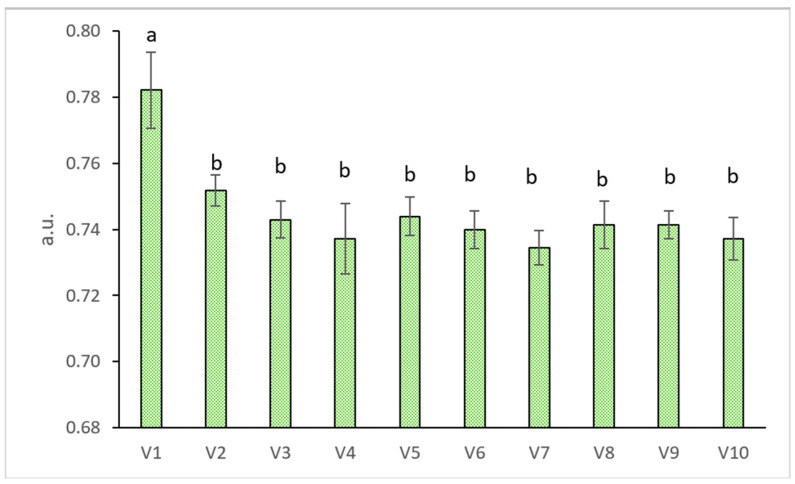
Chlorophyll fluorescence (ΦPSII—quantum yield of PSII of light adapted leaves) of wheat grass cultivated on pond sediment. The values represent the mean ± standard error. The lowercase letters represent a statistically significant difference (Tukey Test, *p* < 0.05). a.u.: arbitrary units.

The gas exchange and total chlorophyll parameters are presented in Table 4. Physiological parameters were most affected in V10 (CS3 100%). The Ci dropped significantly in all variants except V2 (CS1 50% + CP 50%) and V4 (CS1 100%). Transpiration (E) and Gs increased significantly at V6 (CS2 75% + CP 25%) compared to V1 (control) by 62% and 30%. Photosynthesis (A) was the highest at V6, however without a significant difference compared with V1, while at V10 it decreased by 40% compared with the control variant. Regarding the chlorophyll content, there were no significant differences between the variants. Regarding chlorophyll fluorescence (Figure 1), ΦPSII parameter (quantum yield of PSII of light adapted leaves) decreased significantly in all variants compared with the control variant.

### 2.5. Wheat Grass Juice Quality

To evaluate the quality of wheat grass juice depending on the sediment application, the total content of phenols and flavonoids and the antioxidant activity were quantified (Table 5). The total phenol content increased significantly in variants V7 (CS2 100%) and V8 (CS3 50% + CP 50%) compared to variant V1 (control) by 14% and 10%, respectively, and decreased in V3 (CS1 75% + CP 25%) and V9 (CS3 75% + CP 25%) by 9% and 14%, respectively. The flavonoid content decreased significantly in V2 (CS1 50% + CP 25%) and V3 by 19% and 20%, respectively. The highest flavonoid content was recorded in V7, however the difference from V1 was not significant. In terms of antioxidant activity, it increased in V4 (CS1 100%), V5 (CS2 50% + CP 50%), V6 (CS2 75% + CP 25%), and V7 and decreased in V2, V3, and V9 compared to V1.

**Figure 2:**
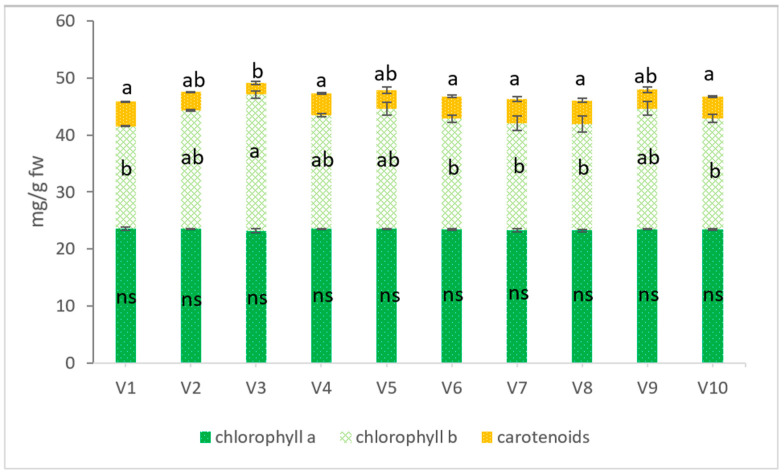
Chlorophyll a, chlorophyll b, and carotenoids in wheatgrass juice. The values represent the mean ± standard error. The lowercase letters represent statistically significant differences according to the Tukey Test, *p* < 0.05. ns: not significant.

The chlorophyll content of wheatgrass juice is shown in Figure 2. No significant differences were recorded in the content of chlorophyll a. However, chlorophyll b, increased significantly with 32% in V3 (CS1 75% + CP 25%) compared with V1 (control). This variant also registered a significant decrease of carotenoid content at 52%.

### 2.6. Chemical Composition of Leachate

The chemical composition of the leachate resulting from the pots used in the experiment is shown in Table 6. In general, the pH was alkaline, with values between 7.73 and 8.37. The nitrate content of the leachate decreased by up to 60% at V3 (CS1 75% + CP 25%) and V4 (CS1 100%), however, the nitrate content increased significantly (*p* < 0.05) with up to 225% at V9 (CS3 75% + CP 25%. The lowest NO_2_ content was recorded in control, while the highest value was obtained at V8 (CS3 50% + CP 50%). The NH_3_ content was low, ranging from 0.02 in V3 and V10 (CS3 100%) to 0.14 in V8.

## 3. Discussion

The objective of this study was to test the effects of a dried aquaculture sediment, from a common carp cultivation pond, on the growth and physiology of potted wheat grass and the quality of the juice obtained from wheat grass. Cypriniculture (cultivation of common carp) occupation is about 8000 years old and is now widespread globally [21]. The traditional ponds used for carp aquaculture represent aquatic ecosystems in which, besides the species of interest in—the carp, there are other populations of plants, animals, and microorganisms, with functions as producers, consumers, and decomposers, between which trophic relations had been established [22]. Sediments from aquaculture consists mainly of fish feces, uneaten food, organic matter, and mineral elements resulting from the decomposition of aquatic organisms [23]. Depending on the type of production, such as aquaculture in ponds or recirculating systems, the physicochemical composition of sediments can vary greatly [24]. In this study, the sun-dried sediments from a carp (*Cyprinus carpio*) growing pond were tested as a nutrient source for wheatgrass production. Physico-chemical analyzes of pond sediments showed an alkaline pH, which is higher compared to other studies, while the organic matter content was relatively low. The pH of sediments can be influenced by numerous factors, such as the quality of the water and the geological substrate, season, the feed used, the productivity of the pond, etc. [25]. Due to the fact that the oxygen content is low in the sediment because of the lack of air spaces, organic material tends to accumulate [1]. The organic matter content was between 5.5 and 6.4%, which are relatively low values compared with the values reported in previous studies [1,2]. This may be due to the sun drying treatment, which may speed up the transformation of organic material into humus (3.16–3.62) and N (0.16–0.19). Regarding the content of P and K, average values were registered (64–118 and 235–276, respectively), while the content of heavy metals was low compared to other studies [2]. Moreover, the Cd concentration in the sediment was below the detection limit of the AAS instrument. These elements come mainly from unconsumed feed and feces, however also from the remains of vegetation and other decomposed organisms as well as from the geological substrate.

The total coliform content in sediment was between 760 and 1536 cfu/g. Sediments, unlike water, ensure a more favorable climate for the development of coliforms due to the presence of organic matter and shelter from predators. The content of coliforms in sediments can vary spatio-temporally, which is why their monitoring is necessary when sediments are used as fertilizers. Harbi (2003) [26], identified a higher content of total coliforms (3.2 × 10^5^–2.88 × 10^7^) in sediments from hybrid tilapia culture compared to this study.

In this study, wheat plants had a relative uniform growth, with no significant differences in plant height, leaf area, and plant fresh production depending on the sediment application rates. However, wheatgrass juice production was highest in the control and decreased by up to 20% at V10. The sediments in the carp growing pond have a moderate content of macronutrients (N, P, and K) and organic matter, which led to normal plant growth in most variants. Small decreases in plant height, leaf area, and juice production were recorded at variant V10 (CS3 100% pond sediment). In general, the sediments have a high density and are free of porosity, which could negatively influence the growth of the plants as in V10 [1]. For this reason, sediments are generally used in a mixture with different materials such as compost, to improve their physical and chemical properties and subsequently to ensure optimal plant growth [11].

Regarding the effect of sediments on wheat physiology, a decrease of up to 40% in photosynthesis at V10 compared with V1 (control) was found, concomitantly with decreases in the transpiration rate, substomatal carbon dioxide, and the stomatal conductance. The gas exchange parameters enumerated above were lower at the same variant (V10) in which the morphological and production parameters decreased compared with the control. The pigment content and green color (a) were lower in V10 compared with those in the control. Moreover, the chlorophyll fluorescence decreased in all variants compared with that in the control. These variations could be caused by the physico-chemical properties of the sediments, which was the only source of nutrients [8].

From a biochemical point of view, wheat grass juice is a complex solution with proven nutritional and therapeutic properties. Fortuna et al. (2018) [19] highlighted rich mineral content in wheatgrass juice (C, N, O, Na, Mg, Si, Cl, K, and Ca) and different types of chlorophyll (pheophytin a, hydroxychlorophyll a, chlorophyll a, and chlorophyll b). Moreover, the same study showed that the content of phenols can vary from 706 to 818 mg/mL of gallic acid and that of flavonoids from 346 to 809 mg/mL of quercetin, and the antioxidant activity can be between 30% and 48% (DPPH) [19]. However, the nutritional and therapeutic value of plants can vary depending on the species and variety [27]. The sediments used in this study as a nutrient source for wheat influenced the quality of wheatgrass juice. For instance, the content of total phenols, flavonoids, and antioxidant activity increased the most in V7 (CS2 100% pond sediment) compared with the control variant, while in other variants these metabolites decreased. Phenols and flavonoids are products of secondary metabolism and are defensive plant chemicals against ultraviolet radiation and herbivores. Thus, plants synthesize these compounds in larger quantities especially when under stress conditions [28,29]. Another factor that influences the synthesis of these compounds is the chemical composition of the substrate. For example, in the case of a soils with low organic matter content, the rosmarinic acid content increased considerably compared to the plants grown on municipal sewage sludge [30]. Wheat grass juice did not show major variations in the content of chlorophyll a, instead it had a significant increase in the content of chlorophyll b at V3 (CS1 75% + CP 25%) and a decrease in carotenoids at the same variant. Chlorophyll plays an essential role in photosynthesis, its synthesis being directly dependent on the N content of the substrate [31]. In general, the sediments used in this study had a relatively high content of organic matter and N, allowing the growth of wheat in good conditions as well as increased chlorophyll synthesis. Carotenoids on the other hand have a role in protecting plants against oxidative stress [32], and the lower content of V3 is correlated with a lower value of antioxidant activity in the same variant.

In this study, the leachate obtained from the studied substrates had an alkaline pH between 7.73 and 8.37. Regarding the nitrate content, the lowest values were recorded at V3 and V4 (CS1 100% pond sediment), 60% lower than in the control. At the same time, at V7, V8 (CS3 50% + CP 50%), and V9 (CS3 75% + CP 25%), values exceeding the maximum limit of 50 mg/L according to Directive 91/271/EEC were registered. Relatively low values were recorded for NO_2_ and NH_3_ content. In general, the content of nitrites and nitrates in groundwater can vary from 1 to 150 mg/L. Pollution with N compounds can cause serious environmental problems such as eutrophication of water or contamination of surface or groundwater causing health problems because compounds such as nitrates and nitrites are known to be carcinogenic [33]. In general, this type of pollution is caused by runoff from intensive field crops and animal farms, or from some industrial activities. Improper storage of sediment or sludge can also contribute to this type of pollution [34]. In this context, it is important to monitor these compounds when sediments are used for plant cultivation to avoid environmental contamination.

Considering that sediment removal is costly, $20,000/ha [35], and potting soil cost is 1–5$ (10 L), the sale of the sediment as a growing substrate for plants demonstrates that this residue can be transformed into a value-added product.

## 4. Materials and Methods

### 4.1. Sediment Collection

The sediment was collected from a commune carp growing pond, which belongs to the Research and Development Station for Aquaculture and Aquatic Ecology, Alexandru Ioan Cuza University, Iasi, Romania. The pond is 25 m wide and 100 m long. The pond was emptied of water and allowed to dry for 30 days. Then, the pond was divided into three collection stations along the length of the basin (CS1—1 m from the shore, CS2—6.25 m from the shore, and CS3—12.5 m from the shore) and five large samples of pond sediment were collected from each station. The sun dried pond sediment collected from three collection stations (CS1, CS2, and CS3) was crushed through a 0.5-cm mesh sieve and used to prepare the mixtures (*v*/*v*) with commercial peat (CP) for the cultivation of wheat resulting in 10 treatments: (V1) CP 100% (control); (V2) CS1 50% + CP 50%; (V3) CS1 75% + CP 25%; (V4) CS1 100%; (V5) CS2 50% + CP 50%; (V6) CS2 75% + CP 25%; (V7) CS2 100%; (V8) CS3 50% + CP 50%; (V9) CS3 75% + CP 25%; and (V10) CS3 100%.

### 4.2. Sediment Analysis

The pond sediment samples were air dried and passed through a 2-mm sieve to obtain the fraction used for each analysis and then stored at 4 °C. Sediment pH was determined in water using an electronic pH meter with a glass electrode (WTW pH 3320, GmbH, Weilheim, Germany). Organic matter was determined according to the wet oxidation method (Walkley and Black); the available N was measured by the Kjeldahl method; while for *p*, the Olsen extractant method was used, following the standard methods according to Soil Studies Development Methodology delivered by the National Institute of Research and Development in Soil Science, Agrochemistry, and Environment [36]. Available K (extracted with neutral 1N NH4OAc) was determined using a high-resolution continuum source, atomic adsorption spectrometer (ContrAA 700, Analytik, Jena, Germany) equipped with a xenon short lamp with a UV arc in hot spot mode and a high-resolution echelle grating monochromator. The flame was generated using an air-acetylene mixture with 99.95% purity.

### 4.3. Elemental Analysis of Sediment

The analysis of minerals was performed at the National Research and Development Institute for Animal Biology and Nutrition IBNA Balotesti. The concentration of the chemical elements was determined according to the atomic absorption spectroscopy method presented in Regulation (EC) no. 152/2009/SR EN ISO 6869: 2002 (Cu, Fe, Mn, Zn), and SR EN 15550: 2008 (Pb and Cd). In brief, the sample was mixed with 65% HNO_3_: 30% H_2_O_2_ (5:2, *v*/*v*) and digested under pressure using a microwave oven (8 min: 130 °C, 5 min: 155 °C, 12 min: 170 °C, 800 W); the solution of the sample was aspirated in the flame of an atomic absorption spectrophotometer with double beam and background correction and the radiation absorption was measured at the wavelength specific to the analyzed element (SOLAAR M6 Dual Zeeman Comfort, Thermo Electron Corp., Cambridge, UK) [37].

### 4.4. Microbiological Analysis of Sediment

Total coliforms and *E. coli* were determined at a National Research and Development Institute for Animal Biology and Nutrition IBNA Balotesti according to the methods SR EN ISO 4831/2006 UFC/g 1020, 1536, 760 E coli SR EN ISO 7251/2006.

The chemical and microbiological characterization of the sediment is presented in Table 1.

### 4.5. Chemical Composition of Sediment Leachate

The pH was measured with Hach HQ11d (Hach Company, Loveland, CO, USA) portable multiparameter, while ammonia, nitrites, and nitrates were assessed with Hanna Iris HI801 Spectrophotometer and Hanna reagent kits (Hanna Instruments, Salaj, Romania).

### 4.6. Cultivation of Wheat Grass

Wheat (*Triticum aestivum*) seeds of Glosa cultivar were germinated and grown directly in black plastic pots with a volume of 1 L, filled with the investigated substrates, each pot containing 100 wheat seeds. For each experimental variant, 9 pots were used. The plants were germinated in the dark for 3 days after which they were taken to natural light and grown for 15 days. Natural light was supplemented with fluorescent tubes (4800 K) 12 h/day. The pots were watered daily with distilled water at 250 mL/pot. The average temperature day/night during the growing period was 27/20 °C.

### 4.7. Fresh Yield and Morphological Parameters of Wheat Grass

At the end of the experiment, fresh biomass was assessed by weighing each plant at an analytical balance and expressed in g. The height of the plants was measured with a ruler and expressed in cm. For each treatment, 10 plants per pot from three pots were measured.

### 4.8. Leaf Area of Wheat Grass

Leaf area was measured with LI-3100C Area Meter, LI-COR, (Lincoln, NE, USA), and expressed in mm^2^. For each treatment, 10 plants per pot from three pots were measured.

### 4.9. The Color of Wheatgrass

Color was measured with the MiniScan XE Plus colorimeter (HunterLab, Reston, VA, USA). The device was calibrated with black and white tiles and the parameters L, a, and b were determined.

L * (lightness to darkness, 100 to 0), a * (redness to greenness, 0 to 100 = red; −80 to 0 = green), and b* (yellowness and blueness, 0 to 70 = yellow; −100 to 0 = blue). L * (lightness—darkness), a * (redness—greenness), and b * (yellowness—blueness).

### 4.10. Total Chlorophyll Content of Wheat Grass

Total chlorophyll content was measured with CCM 200 (chlorophyll content meter), a non-destructive portable device produced by ADC Bioscientific Ltd., Hoddesdon, UK, which measures total chlorophyll and expresses it in CCI units (Chlorophyll Content Index) [38].

### 4.11. Gas Exchange Measurement of Wheat Grass

The gas exchange parameters, photosynthesis, and transpiration were measured with a portable compact system LCI Bioscientific UK Ltd., with a Narrow Leaf Chamber, with an area of 5.8 cm^2^, between 9–10 a.m. [38].

### 4.12. Chlorophyll Fluorescence of Wheat Grass

A chlorophyll meter FMS2, Hansa Tech Ltd., Hoddesdon, UK was used to measure ΦPSII—quantum yield of PSII of light adapted leaves [39].

### 4.13. Wheat Grass Juice Extraction

The plants were cut 1 cm above the substrate, weighed, and the juice was extracted with a juicer by cold pressing.

### 4.14. Chlorophyll Content in Wheat Grass Juice

Assimilatory pigments were extracted in acetone (80%) from ground leaves (0.1 g) and the optical density was read at 470, 646, and 663 nm. Pigment content was calculated using equations described in Wellburn (1994) [40].

### 4.15. Total Phenolic, Total Flavonoid, and Antioxidant Activity of Wheat Grass Juice

The total phenolic and flavonoid content was determined according to Lobiuc et al. (2017) [41]. Extracts (5% *w*/*v*) were prepared from wheat grass juice in methanol (80%) for 24 h at room temperature. Results were expressed as the gallic acid equivalent per ml of fresh weight (µg/mL GAE f.w.) for total phenolic content, and in quercetin equivalent per ml of fresh weight (µg QE f.w.).

Antioxidant activity of WGJ extracts was determined according to Teliban et al. (2020) [42] by the discoloration of DPPH (2.2-diphenyl-1-picrylhydrazyl, Sigma, Schnelldorf, Germany). Results were expressed as % inhibition of DPPH.

### 4.16. Statistical Analyses of Data

The software used for data processing was IBM SPSS v20 (IBM Corp, Armonk, NY, USA), and results were presented as means ± standard errors. The differences between variants were tested by ANOVA (*p* < 0.05) followed by a Levene test for homogeneity of variances and a Tukey multiple comparison post hoc test [43].

## 5. Conclusions

In this study, the effects of sediments from the carp pond on wheat grass production and physiology and the quality of wheat grass juice were tested. The sediments had a slightly alkaline pH, and a relatively rich content of nutrients. The content of heavy metals was below the maximum allowed for use in agriculture and the content of total coliforms was relatively low. The growth of the wheat grass on the pond sediment was relatively uniform. The morphological parameters had values close to the control variant while production increased in some variants. Some physiological parameters were affected. The addition of pond sediment to the growth medium had a negative effect on wheat grass juice yield. Wheat grass juice had a rich content of flavonoids, phenols, and chlorophyll and increased antioxidant activity. We can conclude that the dried pond sediment in combination with peat could be safely used for the cultivation of wheat grass. This study demonstrates that the aquaculture sediment could be transformed into a value-added product and the results are relevant for the sustainable management of this residue.

## Figures and Tables

**Table 1:** The chemical and microbiological composition of the commune carp pond sediments.

Sampling Station	Collection Station 1	Collection Station 2	Collection Station 3
pH	8.06 ^a^ ± 0.05	7.68 ^b^ ± 0.05	7.67 ^b^ ± 0.04
Humus%	3.31 ^a,b^ ± 0.04	3.16 ^b^ ± 0.1	3.62 ^a^ ± 0.07
Organic matter%	6.43 ^a^ ± 0.01	5.57 ^c^ ± 0.02	6.26 ^b^ ± 0.03
N total%	0.16 ^b^ ± 0	0.16 ^b^ ± 0.00	0.19 ^a^ ± 0.00
P mg/kg	118.67 ^a^ ± 3.28	64.33 ^b^ ± 2.03	66.33 ^b^ ± 1.33
K mg/kg	276.33 ^a^ ± 2.6	235.67 ^c^ ± 1.76	250.67 ^b^ ± 2.03
Cu mg/kg	28.27 ^ns^ ± 0.05	27.29 ^ns^ ± 0.05	28.13 ^ns^ ± 0.05
Fe mg/kg	33,695.62 ^ns^ ± 0.05	33,083.57 ^ns^ ± 0.05	34,609.12 ^ns^ ± 0.05
Mn mg/kg	455.92 ^ns^ ± 0.05	448.52 ^ns^ ± 0.05	449.53 ^ns^ ± 0.05
Zn mg/kg	82.67 ^ns^ ± 0.05	81.41 ^ns^ ± 0.05	84.11 ^ns^ ± 0.05
Pb mg/kg	10.89 ^ns^ ± 0.05	10.58 ^ns^ ± 0.05	11.04 ^ns^ ± 0.05
Cd mg/kg	bdl	bdl	bdl
Total coliforms cfu/g	1020	1536	760
*E. coli* cfu/g	nd	nd	nd

The values represent the mean ± standard error. The lowercase letters represent a statistically significant difference (Tukey Test, *p* ˂ 0.05). bdl: below detection limit, nd: not detected, ns: not significant.

**Table 2:** Morphology of wheat grass and yield of wheat grass juice cultivated on pond sediment.

Treatment	Plant Height (cm)	Leaf Area (mm^2^)	Plant Biomass (g/Plant)	Wheat Grass Juice Yield (g/100 g)
V1	17.53 ^ns^ ± 0.61	135.61 ^ns^ ± 7.31	3.93 ^ns^ ± 0.19	58.00 ^b^ ± 1.16
V2	18.06 ^ns^ ± 0.44	121.12 ^ns^ ± 10.88	3.39 ^ns^ ± 0.31	50.14 ^b,c,d^ ± 1.04
V3	18.72 ^ns^ ± 0.48	128.32 ^ns^ ± 2.15	3.82 ^ns^ ± 0.01	48.34 ^c,d^ ± 0.88
V4	19.22 ^ns^ ± 0.43	129.17 ^ns^ ± 2.34	3.97 ^ns^ ± 0.07	55.54 ^a,b,c^ ± 1.08
V5	18.06 ^ns^ ± 0.57	116.93 ^ns^ ± 14.35	3.57 ^ns^ ± 0.42	50.00 ^b,c,d^ ± 1.16
V6	18.06 ^ns^ ± 0.49	139.21 ^ns^ ± 3.19	4.26 ^ns^ ± 0.07	54.00 ^a,b,c^ ± 1.16
V7	17.38 ^ns^ ± 0.42	116.44 ^ns^ ± 5.78	3.55 ^ns^ ± 0.20	56.80 ^a,b^ ± 1.34
V8	19.03 ^ns^ ± 0.69	126.86 ^ns^ ± 12.02	3.83 ^ns^ ± 0.12	52.34 ^a,b,c,d^ ± 0.88
V9	18.81 ^ns^ ± 0.56	110.26 ^ns^ ± 8.83	3.51 ^ns^ ± 0.27	56.34 ^a,b^ ± 1.46
V10	16.94 ^ns^ ± 0.44	109.67 ^ns^ ± 4.10	3.47 ^ns^ ± 0.09	46.00 ^d^ ± 3.06

The values represent the mean ± standard error. The lowercase letters represent a statistically significant difference (Tukey Test, *p* < 0.05), ns: not significant.

**Table 3:** Color of wheat grass cultivated on pond sediment.

Treatment	L	a	b
V1	37.81 ^ns^ ± 0.84	−9.11 ^a,b^ ± 0.2	18.11 ^ns^ ± 0.87
V2	34.75 ^ns^ ± 1.26	−8.59 ^a,b^ ± 0.19	17.19 ^ns^ ± 0.44
V3	36.62 ^ns^ ± 0.45	−8.64 ^a,b^ ± 0.08	16.89 ^ns^ ± 0.28
V4	38.56 ^ns^ ± 0.74	−9.27 ^b^ ± 0.05	18.91 ^ns^ ± 0.09
V5	37.33 ^ns^ ± 0.41	−9.03 ^a,b^ ± 0.07	18.49 ^ns^ ± 0.27
V6	38.47 ^ns^ ± 0.49	−9.04 ^a,b^ ± 0.05	17.96 ^ns^ ± 0.39
V7	35.06 ^ns^ ± 0.43	−8.48 ^a^ ± 0.07	17.09 ^ns^ ± 0.19
V8	36.7 ^ns^ ± 1.21	−8.8 ^a,b^ ± 0.22	17.37 ^ns^ ± 0.93
V9	36.86 ^ns^ ± 0.35	−8.79 ^a,b^ ± 0.09	17.23 ^ns^ ± 0.13
V10	35.97 ^ns^ ± 1.18	−8.43 ^a^ ± 0.22	16.44 ^ns^ ± 0.95

The values represent the mean ± standard error. The lowercase letters represent a statistically significant difference (Tukey Test, *p* < 0.05), ns: not significant. L: lightness—darkness, a: redness—greenness, and b: yellowness—blueness.

**Table 4:** Gas exchange parameters and total chlorophyll content of wheat grass cultivated on pond sediment.

Treatment	Ci	E	Gs	A	CCI
V1	378.97 ^a^ ± 1.66	1.35 ^b,c^ ± 0.03	0.10 ^a,b,c^ ± 0.00	1.47 ^a^ ± 0.07	1.54 ^ns^ ± 0.14
V2	373.13 ^a^ ± 1.46	1.11 ^b,c^ ± 0.02	0.07 ^b,c^ ± 0.00	1.29 ^a^ ± 0.06	1.53 ^ns^ ± 0.20
V3	356.13 ^b,c^ ± 1.47	1.64 ^b^ ± 0.03	0.11 ^a,b^ ± 0.00	1.42 ^a^ ± 0.09	1.48 ^ns^ ± 0.09
V4	371.27 ^a,b^ ± 1.07	1.47 ^b,c^ ± 0.02	0.09 ^a,b,c^ ± 0.00	1.54 ^a^ ± 0.05	1.83 ^ns^ ± 0.23
V5	344.77 ^c^ ± 2.81	0.98 ^c^ ± 0.03	0.05 ^c,d^ ± 0.00	1.44 ^a^ ± 0.11	1.6 ^ns^ ± 0.17
V6	349.9 ^c^ ± 2.18	2.19 ^a^ ± 0.18	0.13 ^a^ ± 0.03	1.66 ^a^ ± 0.11	1.57 ^ns^ ± 0.16
V7	351.43 ^c^ ± 3.30	1.38 ^b,c^ ± 0.02	0.07 ^b,c^ ± 0.00	1.28 ^a^ ± 0.10	1.53 ^ns^ ± 0.14
V8	344.13 ^c^ ± 3.14	1.58 ^b^ ± 0.30	0.05 ^c,d^ ± 0.00	1.36 ^a^ ± 0.14	1.43 ^ns^ ± 0.08
V9	344.97 ^c^ ± 3.71	1.52 ^b,c^ ± 0.15	0.09 ^a,b,c^ ± 0.02	1.38 ^a^ ± 0.13	1.42 ^ns^ ± 0.08
V10	316.63 ^d^ ± 8.27	0.36 ^d^ ± 0.01	0.02 ^d^ ± 0.00	0.82 ^b^ ± 0.09	1.4 ^ns^ ± 0.05

The values represent the mean ± standard error. The lowercase letters represent a statistically significant difference (Tukey Test, *p* < 0.05), ns: not significant. Ci: sub-stomatal CO_2_ concentration—μmol mol−1; E: transpiration—mmol H_2_O m^2^s^−1^; Gs: stomatal conductance—mole CO_2_ m^2^s^−1^, A: photosynthesis—μmol CO_2_ m^2^s^−1^; and total chlorophyll content—CCI.

**Table 5:** Quality of wheat grass juice.

Treatment	Total Phenols Galic Acid µg/mL	Total Flavonoids Quercitin µg/mL	Antioxidant Activity DPPH % Inhibition
V1	2.97 ^c^ ± 0.10	1.77 ^a,b^ ± 0.11	53.35 ^c^ ± 1.49
V2	2.94 ^c,d^ ± 0.10	1.42 ^c^ ± 0.13	47.99 ^e^ ± 0.42
V3	2.69 ^d,e^ ± 0.22	1.41 ^c^ ± 0.11	35.48 ^f^ ± 0.42
V4	3.06 ^b,c^ ± 0.02	1.71 ^a,b^ ± 0.12	60.26 ^a,b^ ± 0.34
V5	2.87 ^c,d^ ± 0.12	1.78 ^a,b^ ± 0.06	60.52 ^a,b^ ± 0.08
V6	2.80 ^c,d,e^ ± 0.05	1.63 ^a,b,c^ ± 0.13	58.04 ^b^ ± 0.23
V7	3.39 ^a^ ± 0.03	1.85 ^a^ ± 0.04	62.70 ^a^ ± 0.51
V8	3.28 ^a,b^ ± 0.02	1.7 ^a,b^ ± 0.02	52.37 ^c,d^ ± 1.64
V9	2.55 ^e^ ± 0.03	1.52 ^b,c^ ± 0.06	48.2 ^d,e^ ± 0.66
V10	2.80 ^c,d,e^ ± 0.04	1.61 ^a,b,c^ ± 0.06	52.88 ^c^ ± 1.11

The values represent the mean ± standard error. The lowercase letters represent a statistically significant difference (Tukey Test, *p* < 0.05), ns: not significant.

**Table 6:** Chemical composition of leachate.

Treatment	pH	NO_3_ (mg/L)	NO_2_ (mg/L)	NH_3_ (mg/L)
V1	7.90 ^b,c,d^ ± 0.06	20.20 ^c^ ± 1.33	0.27 ^d^ ± 0.01	0.03 ^b^ ± 0.00
V2	7.97 ^b^ ± 0.03	19.03 ^d^ ± 1.48	1.19 ^b^ ± 0.00	0.04 ^a,b^ ± 0.00
V3	7.93 ^b,c^ ± 0.03	8.77 ^e^ ± 0.03	0.73 ^c^ ± 0.00	0.02 ^b^ ± 0.00
V4	7.97 ^b^ ± 0.03	8.77 ^e^ ± 0.03	0.28 ^d^ ± 0.01	0.03 ^b^ ± 0.00
V5	7.97 ^b^ ± 0.03	36.63 ^b,c^ ± 1.48	1.19 ^b^ ± 0.00	0.05 ^a,b^ ± 0.00
V6	7.73 ^c,d^ ± 0.03	35.2 ^b,c^ ± 2.54	1.17 ^b^ ± 0.02	0.05 ^a,b^ ± 0.00
V7	7.77 ^c,d^ ± 0.03	52.77 ^a,b^ ± 4.38	1.33 ^a^ ± 0.00	0.05 ^a,b^ ± 0.00
V8	8.37 ^a^ ± 0.03	52.47 ^a,b^ ± 8.97	1.37 ^a^ ± 0.02	0.14 ^a^ ± 0.06
V9	7.77 ^c,d^ ± 0.03	65.97 ^a^ ± 4.38	1.15 ^b^ ± 0.02	0.05 ^a,b^ ± 0.00
V10	7.87 ^b,c,d^ ± 0.03	33.67 ^c^ ± 2.92	1.35 ^a^ ± 0.02	0.02 ^b^ ± 0.00

The values represent the mean ± standard error. The lowercase letters represent statistically significant differences according to the Tukey Test, *p* < 0.05.

## Data Availability

Not applicable.
